# A transient increase in MHC-II^low^ monocytes after experimental infection with *Avibacterium paragallinarum* (serovar B-1) in SPF chickens

**DOI:** 10.1186/s13567-020-00840-7

**Published:** 2020-09-25

**Authors:** Karla Lucía F. Alvarez, Astrid Poma-Acevedo, Manolo Fernández-Díaz

**Affiliations:** Research and Development Laboratories, FARVET, Carretera Panamericana Sur No 766 Km 198.5, Ica, Peru

**Keywords:** *Avibacterium paragallinarum*, infectious coryza, systemic immune response, monocytes, MHC-II

## Abstract

Infectious coryza (IC), an upper respiratory tract disease affecting chickens, is caused by *Avibacterium paragallinarum*. The clinical manifestations of IC include nasal discharge, facial swelling, and lacrimation. This acute disease results in high morbidity and low mortality, while the course of the disease is prolonged and mortality rates are increased in cases with secondary infections. Studies regarding the immune response in infected chickens are scarce, and the local immune response is the focal point of investigation. However, a large body of work has demonstrated that severe infections can impact the systemic immune response. The objective of this study was to evaluate the systemic effects of *Avibacterium paragallinarum* (serovar B-1) infection on immune cells in specific pathogen-free (SPF) chickens. The current study revealed the presence of a transient circulating monocyte population endowed with high phagocytic ability and clear downregulation of major histocompatibility complex class II (MHC-II) surface expression. In human and mouse studies, this monocyte population (identified as tolerant monocytes) has been correlated with a dysfunctional immune response, increasing the risk of secondary infections and mortality. Consistent with this dysfunctional immune response, we demonstrate that B cells from infected chickens produced fewer antibodies than those from control chickens. Moreover, T cells isolated from the peripheral blood of infected chickens had a lower ability to proliferate in response to concanavalin A than those isolated from control chickens. These findings could be related to the severe clinical signs observed in complicated IC caused by the presence of secondary infections.

## Introduction

*Avibacterium paragallinarum (Av. paragallinarum)* is a gram-negative bacterium that has been isolated worldwide and is the aetiological agent of infectious coryza (IC), a disease that contributes to significant economic losses in the poultry industry. Clinical signs of IC include nasal discharge, facial swelling, and lacrimation. This disease is associated with reduced egg production, poor growth, and high morbidity [1, 2]. However, in cases with secondary infections, exacerbation of the clinical signs of the disease and increased mortality are observed [3–6]. The causal agent of the disease is classified by the Kume haemagglutinin serotyping scheme into nine serovars (A-1, A-2, A-3, A-4, B-1, C-1, C-2, C-3, and C-4) [7]. All serovars exhibit tropism related to the upper respiratory tract, including the involvement of nasal passages, infraorbital sinuses, and paranasal sinuses.

The efficiency of the local immune system during infection has been evidenced, as different studies have reported a gradual decrease in the *Av. paragallinarum* abundance in infected tissue [6, 8]. Despite the importance of the immune system in the progression of the disease, few studies have evaluated the role of cells or molecules associated with the immune response. Furthermore, although relevant information was obtained, the studies focused on the local immune response by analysis of nasal tissue samples [9, 10]. However, the prognosis of a disease is a result of a more complicated interaction between the local and systemic immune responses. Studies in mice and humans have demonstrated that local infections or trauma also induce a systemic immune response through the release of damage-associated molecular patterns (DAMP) from damaged or necrotic cells [11, 12]. Moreover, studies in chickens (also demonstrated in humans and mice) have shown that in response to a biological stimulus, macrophages and other cells of the immune system secrete pro- and anti-inflammatory cytokines that are released into the circulatory system to elicit a systemic immune response [13–16]. One of the target populations of these cytokines is monocytes, described as a heterogeneous population in the avian system, which have chemotactic and phagocytic activities and are capable of generating a respiratory burst [17–19]. During infectious or severe inflammatory insult in humans and mice, this population has an important role in compensatory anti-inflammatory response syndrome (CARS), a biological process that is necessary to prevent overwhelming inflammation and avoid organ failure [20, 21]. In addition to the benefits of this orchestrated immune response, a prolonged anti-inflammatory response results in immunoparalysis that is characterized by impaired immunity, which is responsible for a patient’s vulnerability to secondary infections and is a common cause of death [22].

As mentioned above, localized infections can modulate the systemic immune response via diverse mechanisms. Thus, the objective of this study was to evaluate the systemic effects of *Av. paragallinarum* infection on immune cells in specific pathogen-free (SPF) chickens, focusing on the role that mononuclear cells, including monocytes as well as T and B lymphocytes, could play in this important poultry disease.

## Materials and methods

### Birds

Forty-eight SPF 16- or 37-week-old white leghorn chickens were maintained and fed ad libitum. SPF chickens were obtained from Charles River Laboratories (Wilmington, MA, USA), and all chickens were housed in the SPF area of FARVET. The animals were euthanized by a qualified veterinarian using cervical dislocation without anaesthesia, following the American Veterinary Medical Association (AVMA) guidelines.

### Bacterial strain

The well-characterized *Av. paragallinarum* isolate FARPER-107 (serovar B-1) was used in this study [23]. This bacterium was isolated in 2013 from an infectious coryza outbreak on a broiler farm in Arequipa, Peru, as described by Morales and collaborators [23].

### Experimental infection of chickens with *Av. paragallinarum*

As a model for infectious coryza, SPF chickens (16- or 37-week-old) were experimentally infected with *Av. paragallinarum (*serovar B-1) via intrasinus instillation, following the same methodology previously reported [23]. Chickens in the control group were paired according to sex and age. Briefly, *Av. paragallinarum* was inoculated into 7-day-old SPF embryonic eggs. After 24 h, the yolks were collected, and 200 µL of yolk containing 10^6^–10^7^ PFU/mL bacteria were inoculated into each chicken via intrasinus instillation. The clinical signs observed in each chicken were recorded after inoculation and scored according to a previously reported scale as follows: 0, no signs; 1, nasal discharge or slight facial swelling; 2, nasal discharge and moderate facial swelling; 3, abundant nasal discharge and severe facial swelling; and 4, the same signs observed in 3 with the addition of swollen wattles and/or conjunctivitis [24].

### Bacterial detection by PCR

*Av. paragallinarum* was isolated from the nasal cavity on day 4 or 6 post infection using a wet swab and was subsequently transported to the laboratory in ice-cold D-PBS (Sigma-Aldrich, St. Louis, MO, USA, Cat. no. D5773-50L).

Genomic DNA was extracted using a commercial QIAamp MinElute Virus Spin Kit (Qiagen, Hilden, Germany, Cat. no. 57704) according to the manufacturer’s instructions. The target region for PCR included the hypervariable region of the *hmtp210* gene [25], which was amplified with the following primers: forward primer, GGC GAT TTA ACA CGG GAG TC, and reverse primer, TCAT ACC AGA TAA ACG GAT ACCT. A Q5 High-Fidelity PCR Kit (New England Bio Labs, Cat. no. E0555) containing pre-mixed dNTP, Taq polymerase, MgCl_2_ and buffer at optimum concentrations was used for PCR. The thermal cycling steps used for amplification were as follows: 98 °C for 30 s; 35 cycles at 98 °C for 10 s, 58.6 °C for 15 s, and 72 °C for 20 s; and a final step at 72 °C for 2 min. The PCR products, 194 bp in length, were subjected to agarose gel electrophoresis on 2% agarose gels, after which they were visualized and imaged under ultraviolet light (Azure Biosystems, Dublin, CA, USA).

### Isolation and culture of peripheral blood mononuclear cells (PBMC)

Blood was collected from the brachial wing vein into vacutainers (Vacutest Kima, PD, Italy, Cat. no. 12010) containing heparin lithium as an anticoagulant. Mononuclear cells were isolated from blood by density gradient centrifugation (400 × *g* for 30 min at room temperature, without braking*)* using Histopaque-1077 (Sigma-Aldrich, Cat. no. 10771). Thereafter, the cells were washed twice with D-PBS at 300 × *g* for 10 min at room temperature. The cells were resuspended in D-PBS containing 5% foetal bovine serum (FBS; HyClone, GE Healthcare, Logan, UT, USA, Cat. no. SV30180.03) or in cell culture medium and were counted after staining with 0.4% trypan blue solution (Sigma-Aldrich, Cat. no. 93595-50ML).

### Phagocytosis assays

PBMC were seeded in 24-well plates and cultured in serum-free medium (FARMEM, a medium developed in-house at FARVET, proprietary) in the presence of fluorescent beads (Polyscience, Warrington, PA, USA, Cat. no. 17844) pre-diluted in culture medium. Phagocytosis was allowed to proceed for 24 h in a humidified 5% CO_2_ incubator at 41 °C. A control plate was stored at 4 °C. Subsequently, cells were detached with 20 mM EDTA (Calbiochem, San Diego, CA, USA, Cat. no. 324503) at room temperature. Immunostaining was performed at 4 °C using a mouse anti-chicken monocyte/macrophage-PE antibody (detailed in the following paragraph). Finally, cells were incubated at 4 °C with 7-aminoactinomycin D (7-AAD, BD Biosciences, San Jose, CA, USA, Cat. no. 8200–02) and were then analysed by flow cytometry.

### Mononuclear cell proliferation assay

Mononuclear cells isolated as described above were seeded in 96-well, round-bottom plates in the presence or absence of 1 µg/mL concanavalin A (Sigma, Cat. no. C5275-5MG) for 72 h. Next, 25 μM EdU (Thermo Fisher Scientific, Waltham, MA, USA, Cat. no. A10044) reagent was added 16 h before termination of culture. The click reaction and staining were performed according to a previously published protocol [26]. Briefly, cells were labelled with mouse anti-chicken CD4-Alexa Fluor® 647 (detailed in the following paragraph) and fixed with 2% formaldehyde. Subsequently, cells were incubated with the click reaction reagents (6 μM fluorescent azide, 0.3 mM CuSO_4_, and 100 mM ascorbic acid).

### Fluorescent cell staining

To prevent non-specific binding, mononuclear cells were incubated for 10 min at 4 °C with a 1:100 dilution of normal mouse serum (Abcam, Cambridge, MA, USA, Cat. no. ab7486) prior to antibody addition. Thereafter, cells were labelled with directly conjugated monoclonal antibodies (detailed in the following paragraph). All antibodies were diluted to the optimal concentration before use. Cell staining was performed at 4 °C for 20 min. Finally, cells were filtered through a 44 μM-pore size nylon mesh (Merck, Darmstadt, Germany, Cat. no. NY4100010) and analysed by flow cytometry.

### Antibodies and flow cytometry reagents

Mouse anti-chicken CD4-Alexa Fluor® 647 (clone CT-4, Cat. no. 821031), mouse anti-chicken CD8α-PE (clone 3-298, Cat. no. 8405–09), mouse anti-chicken CD3-SPRD (clone CT-3, Cat. no. 8200-13), and mouse anti-chicken Bu-1-FITC (clone AV20, Cat. no. 8395-02) antibodies were purchased from SouthernBiotech (Birmingham, AL, USA). The mouse anti-chicken monocyte/macrophage-PE antibody (clone 5K102, also called clone KUL01, Cat. no. M4520-17) was purchased from USBiological (Salem, MA, USA). Mouse anti-chicken MHC class II-FITC (clone 2G11, Cat. no. ab24882), mouse anti-chicken MHC class I-FITC (clone F21-2, Cat. no. ab24881), and biotin mouse anti-chicken TCR gamma delta (clone TCR1, Cat. no. ab25151) antibodies were purchased from Abcam. Streptavidin Alexa Fluor 750 conjugate (Cat. no. S21384) and Sytox blue dead cell stain (Cat. no. S34857) were purchased from Thermo Fisher Scientific (Waltham, MA, USA). The viability determination reagent 7-AAD (Cat. no. 8200-02), was purchased from BD Biosciences (San Jose, CA, USA).

### Whole blood cell staining

Using a reverse pipetting technique, 100 μL of heparinized blood was transferred into each tube and incubated with the mouse anti-chicken MHC class II-FITC (detailed above) and mouse anti-chicken monocyte/macrophage-PE (detailed above) antibodies for 20 min in the dark at room temperature. After 5 min of incubation with 400 μL of 1X BD FACS lysis solution (BD, Cat. no. 349202), cells were washed twice with 5 mL of PBS containing 5% FBS at 300 × *g* for 5 min at room temperature. Finally, cells were resuspended in 1 mL of D-PBS, and 100 µL of AccuCheck Counting Beads (Thermo Fisher Scientific, Cat. no. PCB100) was added using the reverse pipetting technique. The absolute count was obtained according to the manufacturer’s instructions.

### Flow cytometry

Flow cytometry was performed using a BD FACSMelody flow cytometer (BD Biosciences, USA) equipped with two lasers (488 nm and 635 nm) and a Gallios flow cytometer (Beckman Coulter, USA) equipped with three lasers (488 nm, 635 nm, and 405 nm). The data were analysed using FlowJo software v10.6.1 (BD Biosciences, USA).

### Vaccination against Newcastle disease virus (NDV)

Fourteen 16-week-old SPF chickens were divided equally into 2 groups. One group was infected as described above. Five days post infection, each chicken in each group was vaccinated via the eye-drop route with a 30 μL volume containing 10^7^ 50% egg infective dose (EID_50_/mL) of a non-pathogenic NDV strain (LaSota). On days 0 and 14 post vaccination, serum samples were collected for quantification of anti-NDV antibodies by ELISA.

### ELISA

Antibody titres were determined by indirect ELISA (IDEXX, Westbrook, Maine, USA, Cat. no. 99-09263) following the manufacturer’s instructions. Briefly, 100 μL of each serum sample and 100 μL of the negative and positive control samples were dispensed into wells and incubated for 30 min. The plates were then washed and incubated with 100 μL of conjugate per well for 30 min. Subsequently, the plates were washed, and 100 μL of substrate solution was added per well. The reaction was terminated after the addition of 100 μL stop solution. The plates were read using an Epoch 2 microplate reader (BioTek, USA) at 450 nm. The data were analysed, and the sample-to-positive (S/P) ratio was calculated.

### Statistical analysis

All quantitative data were analysed using GraphPad Prism version 6.1 (GraphPad Software, San Diego, CA, USA). The Mann–Whitney test was utilized to evaluate differences between groups. p ≤ 0.05 was considered to indicate a statistically significant difference.

## Results

### The monocyte population increased in infectious coryza expresses low levels of MHC-II molecules

In this study, we experimentally infected SPF chickens with *Av. paragallinarum (*serovar B-1) via intrasinus instillation to establish a model of infectious coryza. PCR was used either 4 or 6 days post infection to confirm the presence of *Av. paragallinarum* in nasal mucus samples from infected birds, and a scoring system [24] was used to assess the clinical signs observed (Additional file 1). Representative agarose gel electrophoresis results of PCR products amplified with primers corresponding to the sequences of the hypervariable region in the *hmtp210* gene are shown in Additional file 2.

**Figure 1:**
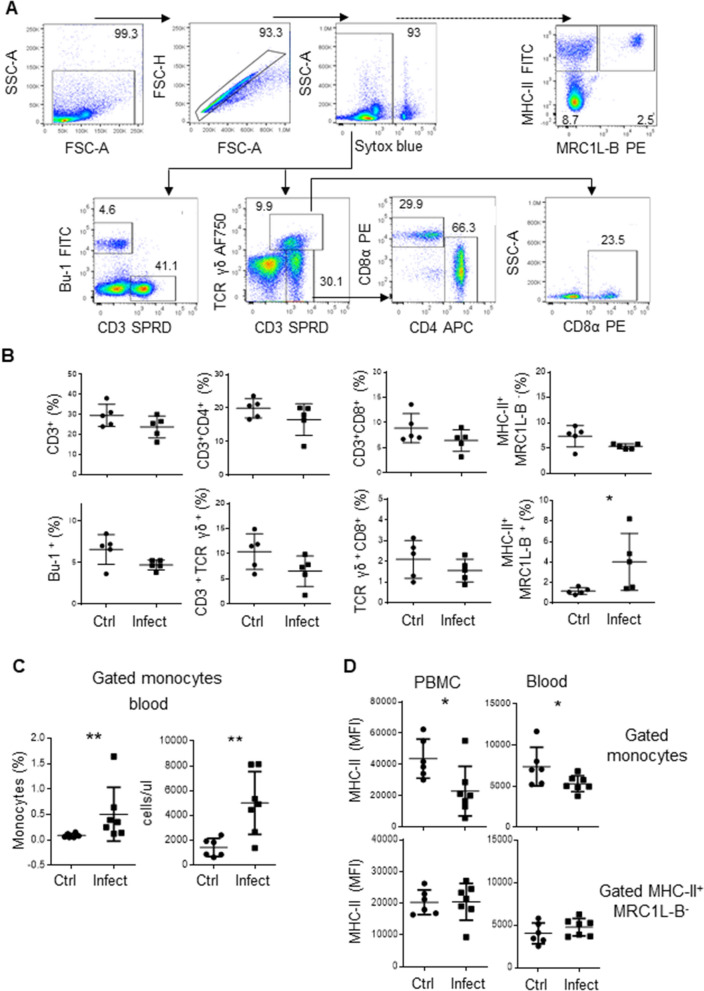
**An increased number of monocytes characterized by low expression levels of MHC-II molecules is observed in chickens infected with Av. paragallinarum**. Mononuclear cells isolated from blood samples were incubated with different antibody cocktails. **A** Gating strategy used to analyse mononuclear cells. We gated out debris, doublets, and dead cells (Sytox blue positive cells), and evaluated the percentages of CD3^+^TCRγδ^+^ cells, CD3^+^TCRγδ^−^ cells, and Bu-1^+^ cells (B cells). The percentages of CD4^+^ and CD8α^+^ cells were evaluated inside the CD3^+^TCRγδ^−^ gate. The percentage of CD8α^+^ cells was also evaluated inside the CD3^+^TCRγδ^+^ gate. The dashed arrow indicates the strategy used to evaluate the populations of monocytes (MRC1L-B^+^) and other APC (MHC-II^+^MRC1L-B^−^) using another antibody cocktail. **B** Quantitative data regarding the percentages of CD3^+^TCRγδ^−^ cells, CD3^+^CD4^+^TCRγδ^−^ cells, CD3^+^CD8α^+^TCRγδ^−^ cells, Bu-1^+^ cells, CD3^+^TCRγδ^+^ cells, CD3^+^TCRγδ^+^CD8α^+^ cells, monocytes and other APC in infected and uninfected animals. The data are presented as percentages of Sytox blue negative cells. Each dot represents an animal. Significant differences are indicated by *p = 0.0317. The results are expressed as the mean ± standard deviation values. A total of 30,000 events per sample were acquired using a Gallios flow cytometer. **C** The figure on the left shows the percentage of monocytes in whole blood. The figure on the right shows the absolute monocyte counts. **D** PBMC or whole blood were used to evaluate MHC-II expression in the monocyte population and MHC-II^+^MRC1L-B^−^ population. The gate used to identify this population is shown in **A**. Each dot represents an animal, and the mean values ± standard deviations are indicated by the bars. Significant differences are indicated by *p = 0.0286 and **p = 0.0023. A total of 30,000 events were acquired for analysis of PBMC, and 300,000 events per sample were acquired for analysis of whole blood samples using a FACSMelody flow cytometer.

To determine the role of immune cells in chickens with infectious coryza, we first measured the percentages of T cells (CD3^+^ TCR αβ^+^, CD3^+^CD4^+^ TCR αβ^+^, CD3^+^CD8^+^ TCR αβ^+^, CD3^+^ TCR γδ^+^, and CD3^+^ CD8^+^ TCR γδ^+^), B cells (Bu-1^+^), monocytes (MRCL1-B^+^), and other antigen-presenting cells (MRC1L-B^−^MHCII^+^) in peripheral blood samples from experimentally infected chickens (n = 5, 37 week-old) and uninfected chickens (n = 5, 37 week-old) using flow cytometry. As shown in Figure 1B, on day 4 post infection, the percentage of monocytes was higher in infected animals than in control animals (4.028 ± 2.8% vs 1.19 ± 0.32%, p = 0.032). No significant differences were observed in other cell populations that were evaluated. To quantitatively determine the number of monocytes, we infected 16-week-old SPF chickens (infected animals: n = 6; control animals: n = 7), and on day 4 post infection, we estimated the absolute number of monocytes based on the acquired bead count using whole blood. As depicted in Figure 1C, the absolute number and percentage of circulating monocytes was higher in infected animals than in control animals (4996.2 ± 2536 cells/μl vs 1412.1 ± 734.8 cells/μl, p = 0.0082 and 0.5 ± 0.01% vs 0.09 ± 0.001%, p = 0.0058, respectively).

It has been reported that chicken monocytes express MHC-II molecules [27, 28]. The importance of this molecule is evidenced by its phylogenetic conservation between species, as well as its crucial role in the functioning of the immune system, including in antigen presentation, rejection of tissue grafts in response to histocompatibility antigens, and resistance to viral diseases [29]. Therefore, using the same experimental group, we evaluated whether MHC-II expression was altered in monocytes from infected animals. As shown in Figure 1D, the mean fluorescence intensity (MFI) of MHC-II was lower in monocytes isolated through density gradient centrifugation from infected animals than in those isolated from control animals (22,890.4 ± 15,891.3 MFI vs 43,655 ± 12,515 MFI, p = 0.021). The same pattern was observed in directly stained samples of whole blood from infected and control animals (5293 ± 967 MFI vs 7781.6 ± 2392.7 MFI, p = 0.035). In this experimental group, similar levels of MHC-II^+^ expression were observed on MRC1L-B^−^MHCII^+^ cells isolated from infected and control animals. The flow cytometry protocol used to analyse the samples of whole blood is shown in Additional file 3.

### MHC-II^low^ monocytes endowed with high phagocytic activity are present during a short period post infection

**Figure 2:**
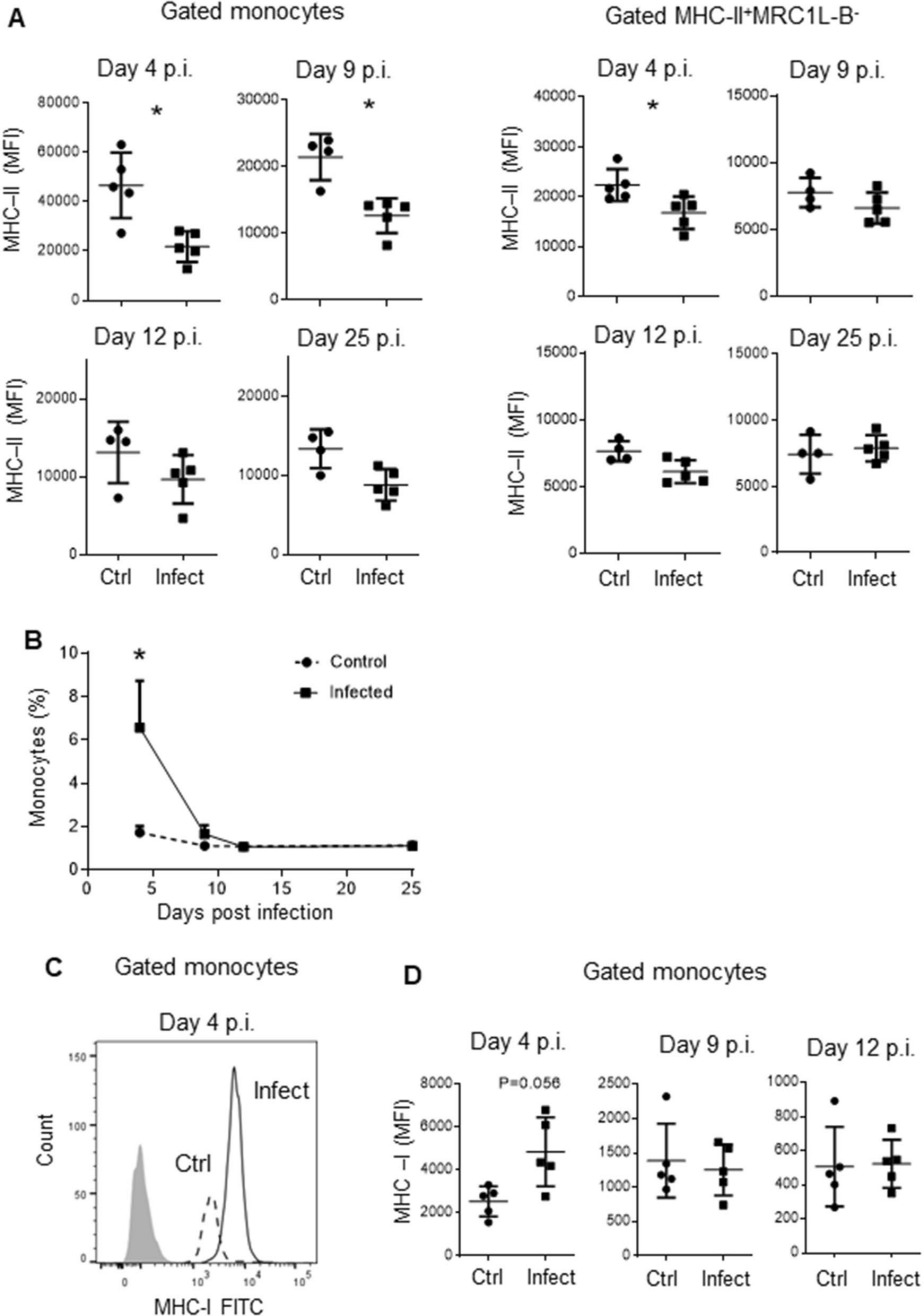
**Evaluation of monocytes and surface expression levels of MHC-II and MHC-I molecules on different days post infection.** Mononuclear cells were isolated from the blood of infected and uninfected animals at 4, 9, 12, and 25 days post infection via density gradient centrifugation using Histopaque-1077. **A** MHC-II surface expression on monocytes and MHC-II^+^MRC1L-B^−^ cells was evaluated using the analysis protocol shown in Figure 1A. Each dot represents an animal. Significant differences are indicated by *p = 0.0159. **B** Each dot represents an experimental group (n = 5) evaluated on different days post infection. Significant differences are indicated by *p = 00,317. **C** Histogram showing the different levels of MHC-I expression on monocytes from infected and uninfected animals. **D** MHC-I surface expression in the monocyte population was evaluated on different days post infection. The results are expressed as the mean ± standard deviation values. A total of 30,000 events per sample were acquired using a Gallios flow cytometer.

To further define the period during which MHC-II^low^ monocytes circulate, we evaluated this peripheral blood mononuclear cell (PBMC) population 4, 9, 12, and 25 days post infection in 37-week-old chickens (infected animals: n = 5, control animals: n = 5). As shown in Figure 2A, B, on day 4 post infection, we observed that MHC-II expression was lower in infected animals than in control animals (21,796.8 ± 6210.2 MFI vs 46,646.8 ± 13,243 MFI, p = 0.016) and that the percentage of monocytes was higher in infected animals (4.6 ± 0.05% vs 1.73 ± 0.01%, p = 0. 032). Nine days post infection, MHC-II expression remained lower in infected animals than in control animals (21,459.3 ± 3473.4 MFI vs 12,660.6 ± 2605.3 MFI, p = 0.016); however, the percentage of monocytes was similar in both groups (1.67 ± 0.9% vs 1.12 ± 0.32%, p = 0.4). No significant differences were observed between the experimental and control groups regarding the percentage of circulating monocytes and MHC-II expression 12 and 25 days post infection. In contrast to the low expression of MHC-II in monocytes from infected animals observed 4 days post infection, the MFI of MHC-I expression on monocytes was slightly higher in infected samples than in control samples (4841 ± 1609 MFI vs 2534 ± 699 MFI, p = 0.05) (Figure 2C, D), while the MHC-I expression values were similar between the groups 12 days post infection (1252 ± 372 MFI vs 1396 ± 539 MFI, p = 0.9). On the other hand, on day 4 post infection, MHC-II expression was lower in MRC1L-B^−^MHCII^+^ cells that were isolated from infected animals compared to those isolated from control animals (16,824.2 ± 3248.1 MFI vs 22,375.4 ± 3195.5 MFI, p = 0,0317) (Figure 2A).

There are reports that some bacteria or viruses can reduce the surface expression of MHC-II molecules as an immune evasion mechanism [30, 31]. Considering that *Av. paragallinarum* has been recovered from naturally sterile sites such as the liver, spleen, and hocks [4], we evaluated the presence of bacterial DNA in lysed PBMC or sorted monocytes via conventional PCR. Although we detected the housekeeping gene GAPDH, we did not detect the presence of bacterial DNA in the PBMC.

**Figure 3:**
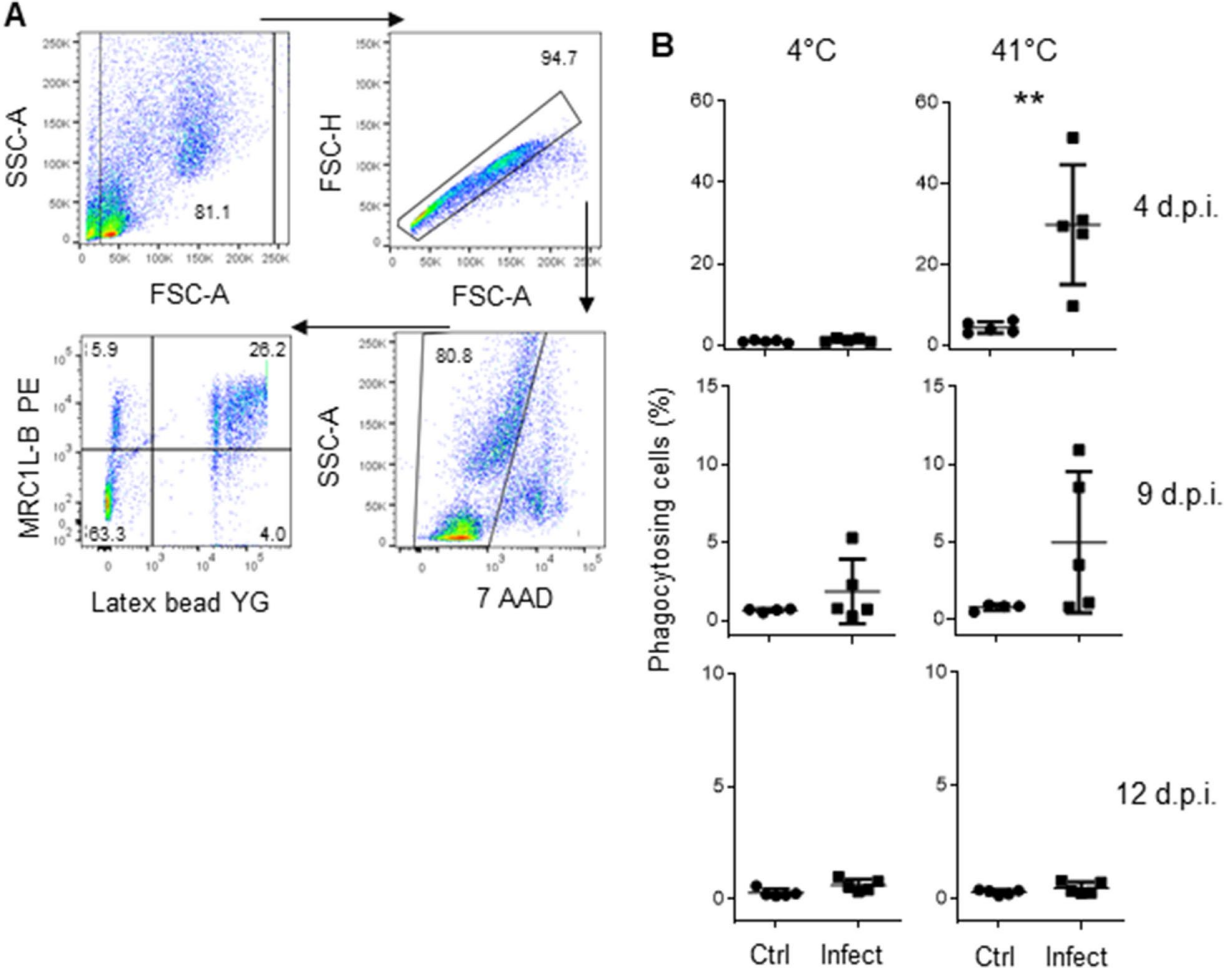
**Monocytes from infected animals exhibit higher phagocytic activity than monocytes from control animals.** Mononuclear cells were isolated from the blood of infected and uninfected animals via density gradient centrifugation using Histopaque-1077 and cultured in the presence of yellow green (YG) fluorescent beads. A duplicate plate was stored at 4 °C as a negative control for phagocytosis. **A** Gating strategy used to analyse mononuclear cells. We gated out debris, doublets, and dead cells (7-AAD-positive cells), and evaluated the percentage of cells that were MRC1L-B^+^YG^+^ double positive. **B** Quantitative data regarding the percentage of monocytes that phagocytized the beads at 41 °C or 4 °C (control). The data are presented as the percentage of 7-AAD-negative cells. Each dot represents an animal. A total of 20,000 events per sample were acquired using a FACSMelody flow cytometer. The results are expressed as the mean ± standard deviation values. Significant differences are indicated by **p = 0.0079.

It has been reported that chicken monocytes are capable of phagocytosis [32]; therefore, we examined whether the phagocytic ability of these monocytes is decreased, increased, or unchanged during infection. To address this question, we cultured PBMC from these chickens for 24 h in the presence of yellow-green (YG) fluorescent beads. To determine that the fluorescence signal originated from internalized beads and not from attached surface beads, a control plate was stored at 4 °C. The flow cytometry protocol used to analyse the phagocytic activity of the monocytes is shown in Figure 3A. Four days post infection, we observed that the percentage of phagocytizing monocytes was higher in samples from infected animals than in samples from control animals (29.84 ± 0.14% vs 4.38 ± 0.01%, p = 0.0079), as shown in Figure 3B. Nine days post infection, the phagocytic activity in both infected and uninfected samples started to return to baseline values (4.96 ± 0.05% vs 1.98 ± 0.002%, p = 0.0952), and phagocytic activity was similar in both groups (0.47 ± 0.002% vs 0.29 ± 0.0012%, p = 0.53) 12 days post infection.

### T cell proliferation and antibody production are affected in infected animals

**Figure 4:**
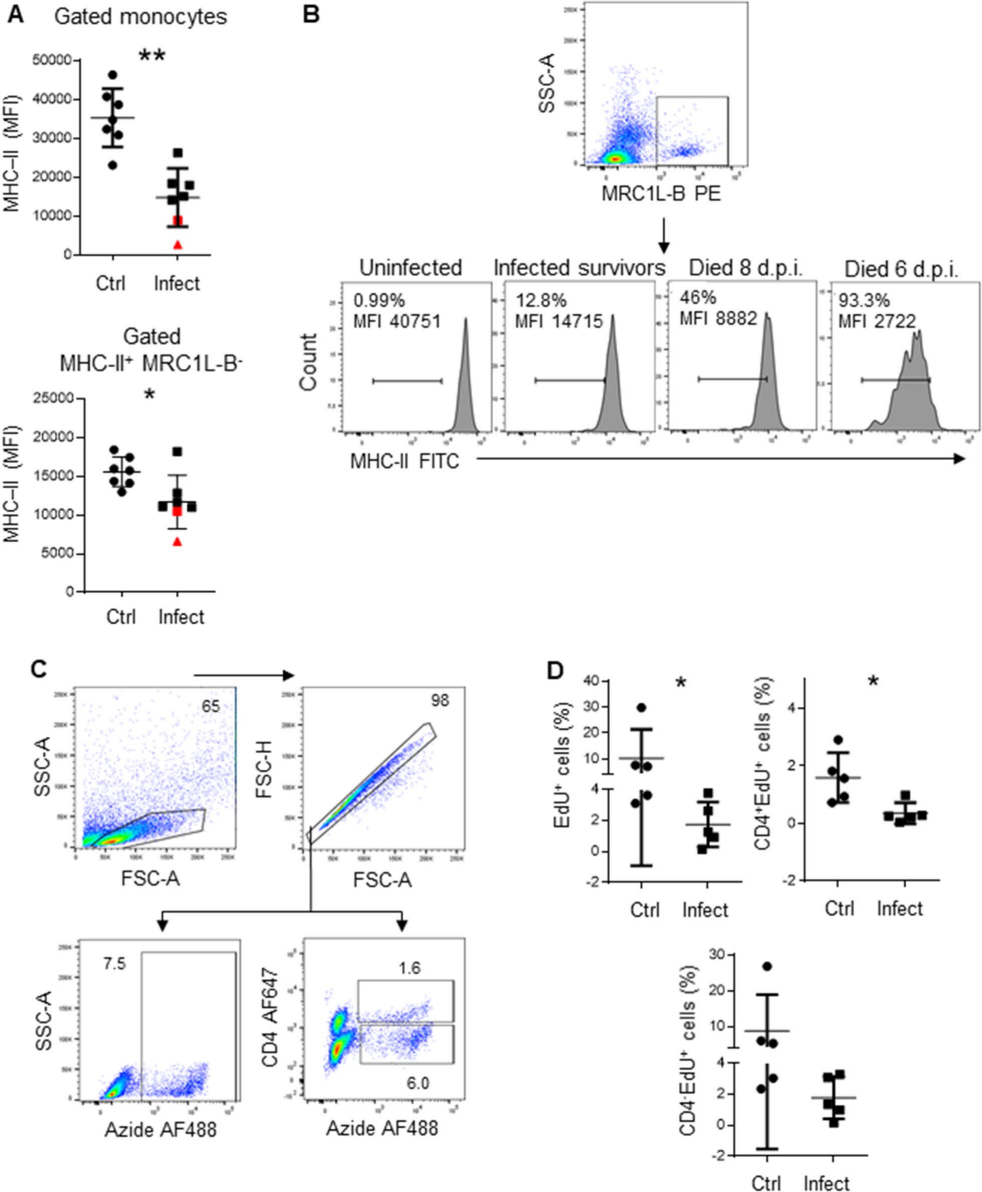
**T lymphocytes isolated from infected animals showed less proliferation than those isolated from control animals.** **A** The MFI of MHC-II expression in monocytes and MHC-II^+^MRC1L-B^−^ cells isolated from infected and uninfected animals. **B** The MFI of MHC-II expression in monocytes and the percentages of MHC-II^low^ monocytes isolated from uninfected animals, infected survivors, and infected non survivors. **C** Gating strategy used to analyse proliferating cells. **D** Left: the percentage of EdU-positive cells within the singlet cells. Right: the percentage of EdU-positive cells within the CD4^+^ population. Bottom: the percentage of EdU-positive cells within the CD4^−^ population. The results are expressed as the mean ± standard deviation values. A total of 50,000 events per sample were acquired using a FACSMelody flow cytometer. Significant differences are indicated by *p = 0.0317.

We also investigated whether lymphocytes from infected animals have the same proliferation ability as lymphocytes from control animals. To achieve this objective, we infected 5 animals with *Av. paragallinarum,* and 5 animals (16-week-old chickens) were used as controls. Four days post infection, we confirmed the presence of MHC-II^low^ monocytes in infected animals, and the results of the EdU proliferation assay revealed that lymphocytes from these animals were less proliferative than lymphocytes from control animals (1.74 ± 1.43% vs 10.21 ± 11.12%, p = 0.0079), as shown in Figure 4A, D. Using antibodies, we observed that this reduction in proliferation was more pronounced for CD4^+^ T cells (0.36 ± 0.36% vs 1.59 ± 0.86%, p = 0.0317) than for CD4^−^ T cells (1.77 ± 1.21% vs 8.7 ± 10.2%, p = 0.095).

In this experimental group, MHC-II expression in monocytes isolated from two infected chickens (denoted with a red triangle and a red square) was lower than expected (Figure 4A, B). Intriguingly, the chicken represented by the red triangle, with an MHC-II MFI of 2722, died 6 days post infection, while the chicken represented by the red square, with an MHC-II MFI of 8882, died two days later. Furthermore, MHC-II^+^MRC1L-B^−^ cells isolated from the chicken represented by the red triangle also exhibited the lowest MHC-II MFI (6636). Based on MHC-II expression, we also observed the presence of two subpopulations of circulating monocytes: MHC-II^low^MRC1L-B^+^ and MHC-II^+^MRC1L-B^+^ cells. The MHC-II^low^MRC1L-B^+^ monocytes accounted for 93.3% and 46% of the monocytes isolated from the chickens that died 6 and 8 days post infection, respectively, while this population accounted for at most 12.8% of the monocytes in the surviving infected animals. In the uninfected animals, this population accounted for at most 1% of the monocytes. We could not correlate the death of the animals to clinical signs of the disease. Although the presence of bacterial DNA was detected in the mucosal samples by PCR, both chickens exhibited only mild facial swelling.

**Figure 5:**
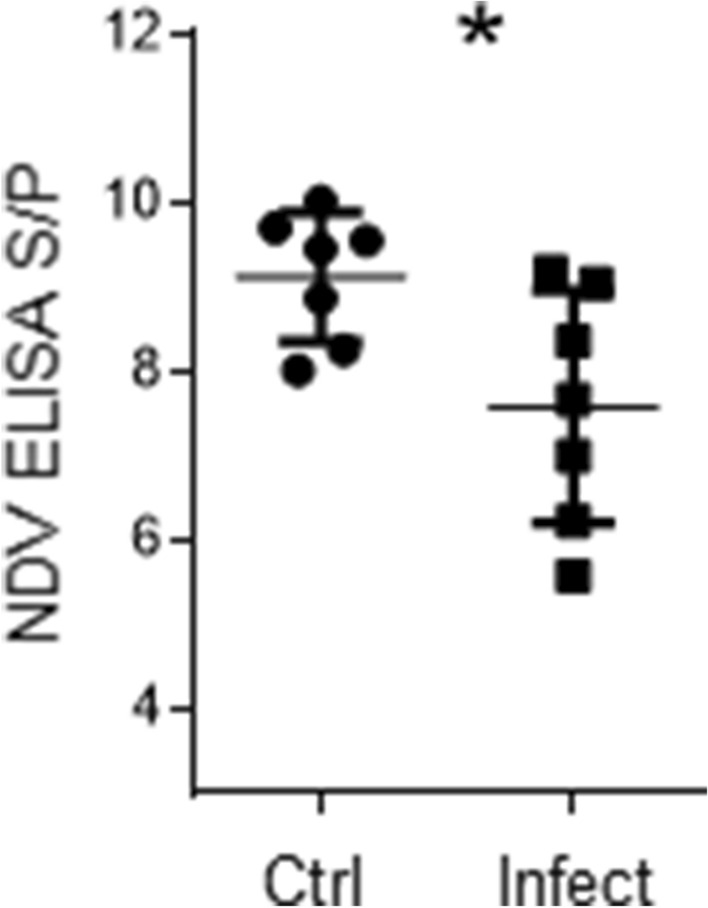
**Lower antibody titres were produced in infected animals than in uninfected animals.** Five days post infection, control and infected animals were inoculated with recombinant Newcastle disease virus (LaSota strain). On the day of vaccination, as well as 14 days post vaccination, serum samples were collected from both experimental groups. Circulating NDV-specific antibodies were assessed by enzyme-linked immunosorbent assay (ELISA). **A** The MFI of MHC-II molecule expression in monocytes and MHC-II^+^MRC1L-B^−^ cells isolated from infected and uninfected animals is shown in Figure 1D. **B** ELISA results are presented as the S/P ratios. Significant differences are indicated by *p = 0.0379. The results are expressed as the mean ± standard deviation values.

Considering that the main function of MHC-II molecules relates to antigen presentation, we investigated whether antibody production was compromised. Therefore, 5 days post infection, chickens were inoculated with the Newcastle disease virus (NDV) vaccine (LaSota strain). As shown in Figure 5, infected animals (16 weeks old) with circulating MHC-II^low^ monocytes and MRC1L-B^−^ cells with a normal MHC-II expression level (Figure 1D) on day 4 post infection produced lower titres of antibodies against NDV than uninfected chickens (16 weeks old), as evaluated on day 14 post vaccination (S/P ratio: 7.6 ± 1.3 vs 9.1 ± 0.76, p = 0.0379).

## Discussion

The immunological response that is essential for defining the severity and physiological outcome of infectious diseases is poorly studied in infectious coryza. Available studies have evaluated only the local immune response by analysis of nasal tissue samples [9, 10]. As a comprehensive overview of the immune system response is lacking, we evaluated the systemic effects of infection with *Av. paragallinarum* on the immune system of SPF chickens.

In the present study, we found that infected chickens developed peripheral blood monocytosis. An increase in the number of monocytes was also observed in chickens that were injected intravenously with lipopolysaccharide (LPS), a major constituent of gram-negative bacteria [33]. Interestingly, we observed that these monocytes were characterized by decreased expression levels of the MHC-II molecule compared to those on monocytes from control animals. Furthermore, on day 4 post infection, we observed a slight increase in MHC-I expression, suggesting that these cells were exposed to a stimulus. To our knowledge, there are no previous reports indicating an association of MHC-II^low^ monocytes with either an effective or a defective immune response in chickens. However, a large body of work has demonstrated that in humans as well as in mice, low MHC-II expression is related to diminished T lymphocyte activation [34, 35]. Moreover, low monocyte HLA-DR expression is used as a robust marker of immunosuppression or immunoparalysis, as the presence of this population has been correlated with an increased risk of secondary infections leading to late morbidity and mortality in critically ill patients admitted for sepsis, trauma or postsurgical complications [36–41]. Regarding infectious coryza, it has been demonstrated that animals infected with *Av. paragallinarum* and then infected with *O. rhinotracheale* 3 days later, showed more severe clinical signs and higher morbidity than chickens infected with *Av. paragallinarum or O. rhinotracheale* alone [23]. Another study showed that simultaneous experimental infection with *Av. paragallinarum* and *Gallibacterium anatis* increased the severity of clinical signs of infectious coryza, resulting in the death of four birds [6]. In field outbreaks, it was demonstrated that *Mycoplasma synoviae* and *Mycoplasma gallisepticum* intensified clinical signs, pathological lesions, and mortality in complicated cases of infectious coryza [3–5]. These studies illustrated that secondary infections can exacerbate the clinical signs of the disease, increasing the morbidity and mortality rates. In the current study, we reported the death of two animals in the infected group. Although these chickens did not exhibit strong clinical signs of the disease, MHC-II^low^ monocytes accounted for more than 45% of the circulating monocytes. Conversely, this population accounted for less than 13% of the circulating monocyte population in infected survivors and less than 1% in control animals. Based on our results and the available literature, we suggest that as in humans, the presence of MHC-II^low^ monocytes in chickens may be related to a dysfunctional immune response, and as a consequence, secondary infections exacerbate the clinical signs of infectious coryza, resulting in increased morbidity and mortality rates. This study also demonstrates the heterogeneity of circulating monocytes (MHC-II^low^ and MHC-II^+^ monocytes) during infection. A recent study demonstrated that under physiological conditions, splenic monocytes can be separated into MHC-II^low^ and MHC-II^high^ populations. Interestingly, MHC-II^low^ monocyte population is increased under inflammatory conditions induced by intraperitoneal LPS injection [42]. On the other hand, Hu and collaborators also demonstrated the monocytes heterogeneity and separated blood monocytes into two subpopulations based on the expression of TIM4 (a receptor that binds to phosphatidylserine) [19]. It would be of great interest to determine whether MHC-II downregulation occurs in both subpopulations or if it is restricted to one population.

By studying the biological activity of the monocytes, we found that monocytes isolated from infected animals have higher phagocytic activity than control monocytes. This observation was consistent with previous reports showing that lipopolysaccharide-tolerant human monocytes demonstrate increased phagocytic ability, although their antigen presentation capacity is impaired due to downregulation of co-stimulatory and MHC-II molecules [35, 43]. The above mentioned results are also in line with studies in mice showing that endotoxin tolerance increases bacterial clearance [44, 45]. Regarding studies on chickens, Sun and collaborators demonstrated that in vitro phagocytosis by monocytes can also be correlated with bacterial clearance under both in vitro and in vivo conditions [46]. Thus, our results suggest that MHC-II^low^ monocytes may contribute to *Av. paragallinarum* clearance; however, future studies evaluating the antigen presentation ability of these cells are necessary.

In the field and in experimental infections, it was observed that effective clearance of *Av. paragallinarum* occurs, as bacteria are eliminated days after initial infection [6, 8]. Confirming the role of immune cells in the in situ inflammatory response, infiltration of lymphocytes and heterophils (the avian counterpart of mammalian neutrophils) into the mucous membranes and lamina propria of the infraorbital sinus was demonstrated in infected animals [47]. Heterophils have the ability to clear bacteria by phagocytosing bacteria, degranulating, and generating an oxidative burst [48], and as demonstrated by Bojesen and collaborators, these cells also mediate local inflammation and tissue necrosis [49]. However, local inflammation cannot predict an inflammatory or anti-inflammatory systemic immune response. Moreover, as demonstrated by other investigators, the functional phenotype of immune cells, such as macrophages, can undergo reversible changes in response to the surrounding environment [50, 51]. This ability was exemplified in cystic fibrosis patients, where the type 1 macrophage (M1) phenotype was the most common in nasal tissue, while circulating monocytes exhibited a tolerant M2 phenotype [35, 52]. This macrophage dichotomy (M1/2) is not established in chickens. However, a recent study provides novel evidence for the existence of these monocyte populations in chickens [42]. That study demonstrated an increase in MRC1L-B^high^MHC-II^low^ splenic cells (possibly M2) under inflammatory conditions (induction via intraperitoneal LPS injection). Moreover, these cells characterized by high phagocytic activity produced lower amounts of inflammatory cytokines (IL-1β, IL-6, and IL-12p40) than MRC1L-B^low^MHC-II^high^ (possibly M1) cells. Based on this information, we believe that although the occurrence of a local inflammatory immune response during *Av. paragallinarum* infection is indicated in previous reports, it is plausible that the circulating MHC-II^low^ monocytes described in the present study could contribute to an anti-inflammatory systemic immune response. Future research is needed to confirm or discard our hypothesis.

It was reported that a systemic anti-inflammatory immune response, termed compensatory anti-inflammatory response syndrome (CARS), acts as a counter-regulatory mechanism for maintaining homeostasis that aims to prevent overwhelming inflammation and is characterized by an impaired cellular immune response [22]. In line with this observation, we also reported that the cellular adaptive immune response could be compromised in infected chickens, as the T lymphocytes isolated from the blood of infected chickens were less proliferative than those isolated from control animals. This difference can be explained by T lymphocyte anergy or by the presence of a circulating suppressor population in cell culture. In the latter case, the activity of this suppressor may be mediated by soluble factors, as we used a TCR-independent antigen to stimulate the cells. Further studies are required to elucidate the mechanism involved in the diminished responses to mitogen stimulation in T lymphocytes. We also demonstrated that the ability of B cells to produce antibodies was lower in infected animals than in control animals, indicating that the humoural immune response was also compromised. In this experimental group, the surface expression level of MHC-II in MRC1L-B^−^ cells (most of which were B cells) from infected and control animals was similar. Strikingly, in other experimental groups, we observed reduced expression of MHC-II not only in the monocyte population but also in other APC (MHC-II^+^MRC1L-B^−^). We cannot attribute this variation between experimental groups to the age of the chickens, as this downregulation was observed in both 16- and 37-week-old chickens, or to the intensity of the clinical signs, as the chickens exhibited mild signs. It is possible that dysregulation of circulating B cells or other APC could be more pronounced and homogeneous in chickens that exhibit severe clinical signs of the disease or are infected with another serovar.

In this study, we generally observed mild signs of the disease. The pathogenicity of *Av. paragallinarum* has been correlated with the serovar, challenge method, host, and dosage [53]. We reproduced IC using an artificial intrasinus instillation method, which is different from the in-contact challenge method (similar to natural infection), and the clinical signs were less persistent and intense in our model than in the in-contact challenge model. Further studies using the in-contact challenge model or farm conditions are necessary to confirm or deny an association between clinical signs and the severity of the impaired systemic immune response. Moreover, as differences in local inflammation between serovars could exist, we cannot predict the reproducibility of the results in chickens infected with another serovar. However, this study revealed novel information regarding the systemic immune response in chickens infected with serovar B-1 that could be used as a reference point in evaluating the immune response elicited by other serovars and gram-negative bacteria.

In summary, based on the low expression of MHC-II on monocytes, reduced antibody production ability, and defective activation of T cells, we suggest that animals infected with *Av. paragallinarum* (serovar B-1) have a dysfunctional immune response that could be related to the severe clinical signs observed in complicated IC. Furthermore, as low MHC-II expression and high phagocytic activity are some of the hallmarks of tolerant monocytes (well characterized in mouse and human studies), we present the first evidence that a population of these cells is present in chickens infected with *Av. paragallinarum* (serovar B-1). The study of the specific molecules and mechanisms involved in the dysregulation of the innate and adaptive immune systems will contribute to the development of novel vaccines and/or therapeutic strategies to control this life-threatening disease.

## Supplementary information

** Additional file 1. Clinical sign scores of the chickens infected with *Av. paragallinarum*** **that were used in this study**. The clinical signs observed were scored according to the following scale: 0, no signs; 1, nasal discharge or slight facial swelling; 2, nasal discharge and moderate facial swelling; 3, abundant nasal discharge and severe facial swelling; and 4, the same signs as 3 with the addition of swollen wattles. (**A**) The clinical signs were recorded in the experimental group of 37-week-old chickens from which mononuclear cells were isolated and treated according to a previously determined protocol, thereby generating the results presented in Figures 1A and B. (**B**) The clinical signs were recorded in the experimental group of 37-week-old chickens from which mononuclear cells were isolated 4, 9, 12, and 25 days post infection and treated according to a previously determined protocol, thereby generating the results presented in Figures 2 and 3. (**C**) The clinical signs were recorded in the experimental group of 16-week-old chickens from which mononuclear cells were isolated and treated according to a previously determined protocol, thereby generating the results presented in Figures 1C, 1D, and 5. (**D**) The clinical signs were recorded in the experimental group of 16-week-old chickens from which mononuclear cells were isolated and treated according to a previously determined protocol, thereby generating the results presented in Figure 4. *: Bacterial DNA detected by PCR; -: animal death.

** Additional file 2. Evaluation of the presence of Av. paragallinarum in mucosal samples from infected animals and uninfected animals by PCR.** Representative agarose gel electrophoresis results for PCR products amplified with primers based on the sequences of the hypervariable region in the *hmtp210* gene were used to identify *Av. paragallinarum*-infected chickens. Line M (marker): 1000 bp DNA ladder, NTC: no-template control, C(+): positive control, and C(−): negative control.

** Additional file 3.** Gating strategy used to analyse whole blood samples.

## Data Availability

The dataset supporting the conclusions of this article is available in the Mendeley data repository. All raw data are also stored on the laboratory server and in cloud storage and will be made available upon request. We have not provided the composition of the FARMEM medium (proprietary); however, this medium will be available at a reasonable cost and in limited quantities to members of the scientific community for non-commercial purposes.

## Abbreviations

AF: Alexa Fluor
*Av. paragallinarum*: *Avibacterium paragallinarum*
CARS: compensatory anti-inflammatory response syndrome
DAMP: damage-associated molecular patterns
ConA: concanavalin A
ChS: chicken Serum
D-PBS: Dulbecco’s phosphate-buffered saline
EDTA: ethylenediamine tetraacetic acid
EdU: 5-Ethynyl-2´-deoxyuridine
FACS: fluorescence-activated cell sorting
FBS: foetal bovine serum
IC: infectious coryza
MHC-I: major histocompatibility complex class I
MHC-II: major histocompatibility complex class II
MFI: mean fluorescence intensity
NDV: Newcastle disease virus
PBMC: peripheral blood mononuclear cells
PE: phycoerythrin
PEG: polyethylene glycol
PFU: plaque forming units
qPCR: quantitative polymerase chain reaction
RPMI: Roswell Park Memorial Institute
7-AAD: 7-Aminoactinomycin D
