# Supplementary material for: A transient increase in MHC-IIlow monocytes after experimental infection with Avibacterium paragallinarum (serovar B-1) in SPF chickens
Source: Vet Res. 2020 Sep 25;51:123. doi: 10.1186/s13567-020-00840-7 (PMC7517641; doi:10.1186/s13567-020-00840-7)
Supplement: Supplementary file 1 — Additional file 1. Clinical sign scores of the chickens infected with Av. paragallinarum that were used in this study. The clinical signs observed were scored according to the following scale: 0, no signs; 1, nasal discharge or slight facial swelling; 2, nasal discharge and moderate facial swelling; 3, abundant nasal discharge and severe facial swelling; and 4, the same signs as 3 with the addition of swollen wattles. (A) The clinical signs were recorded in the experimental group of 37-week-old chickens from which mononuclear cells were isolated and treated according to a previously determined protocol, thereby generating the results presented in Figures 1A and B. (B) The clinical signs were recorded in the experimental group of 37-week-old chickens from which mononuclear cells were isolated 4, 9, 12, and 25 days post infection and treated according to a previously determined protocol, thereby generating the results presented in Figures 2 and 3. (C) The clinical signs were recorded in the experimental group of 16-week-old chickens from which mononuclear cells were isolated and treated according to a previously determined protocol, thereby generating the results presented in Figures 1C, 1D, and 5. (D) The clinical signs were recorded in the experimental group of 16-week-old chickens from which mononuclear cells were isolated and treated according to a previously determined protocol, thereby generating the results presented in Figure 4. *: Bacterial DNA detected by PCR; -: animal death. [file 13567_2020_840_MOESM1_ESM.docx]

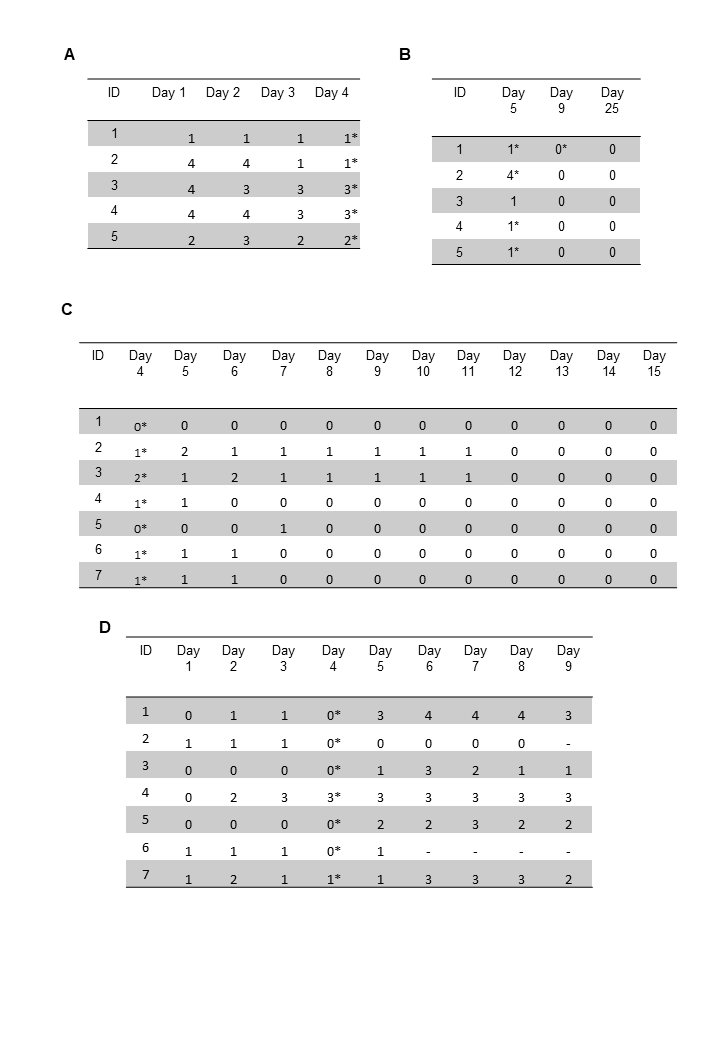


**Additional file 1. Clinical sign scores of the chickens infected with *Av. paragallinarum* that were used in this study.** The clinical signs observed were scored according to the following scale: 0, no signs; 1, nasal discharge or slight facial swelling; 2, nasal discharge and moderate facial swelling; 3, abundant nasal discharge and severe facial swelling; and 4, the same signs as 3 with the addition of swollen wattles. (**A**) The clinical signs were recorded in the experimental group of 37-week-old chickens from which mononuclear cells were isolated and treated according to a previously determined protocol, thereby generating the results presented in Figure 1A and B. (**B**) The clinical signs were recorded in the experimental group of 37-week-old chickens from which mononuclear cells were isolated 4, 9, 12, and 25 days post infection and treated according to a previously determined protocol, thereby generating the results presented in Figures 2 and 3. (**C**) The clinical signs were recorded in the experimental group of 16-week-old chickens from which mononuclear cells were isolated and treated according to a previously determined protocol, thereby generating the results presented in Figures 1C, 1D, and 5. (**D**) The clinical signs were recorded in the experimental group of 16-week-old chickens from which mononuclear cells were isolated and treated according to a previously determined protocol, thereby generating the results presented in Figure 4. *: Bacterial DNA detected by PCR; -: animal death.
