# Supplementary material for: A transient increase in MHC-IIlow monocytes after experimental infection with Avibacterium paragallinarum (serovar B-1) in SPF chickens
Source: Vet Res. 2020 Sep 25;51:123. doi: 10.1186/s13567-020-00840-7 (PMC7517641; doi:10.1186/s13567-020-00840-7)
Supplement: Supplementary file 2 — Additional file 2. Evaluation of the presence of Av. paragallinarum in mucosal samples from infected animals and uninfected animals by PCR. Representative agarose gel electrophoresis results for PCR products amplified with primers based on the sequences of the hypervariable region in the hmtp210 gene were used to identify Av. paragallinarum-infected chickens. Line M (marker): 1000 bp DNA ladder, NTC: no-template control, C(+): positive control, and C(−): negative control. [file 13567_2020_840_MOESM2_ESM.docx]

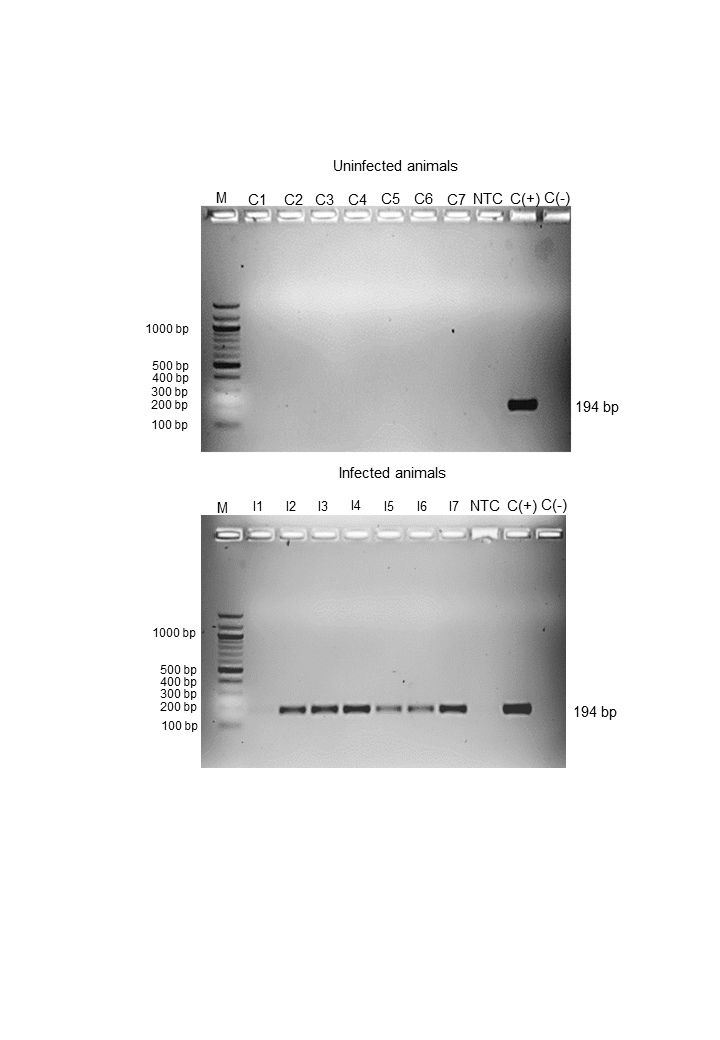


**Additional file 2. Evaluation of the presence of *Av. paragallinarum* in mucosal samples from infected animals and uninfected animals by PCR.** Representative agarose gel electrophoresis results for PCR products amplified with primers based on the sequences of the hypervariable region in the *hmtp210* gene were used to identify *Av. paragallinarum-*infected chickens. Line M (marker): 1000 bp DNA ladder, NTC: no-template control, C(+): positive control, and C(-): negative control.
