# Supplementary figures and images for: A transient increase in MHC-IIlow monocytes after experimental infection with Avibacterium paragallinarum (serovar B-1) in SPF chickens
Source: Vet Res. 2020 Sep 25;51:123. doi: 10.1186/s13567-020-00840-7 (PMC7517641; doi:10.1186/s13567-020-00840-7)

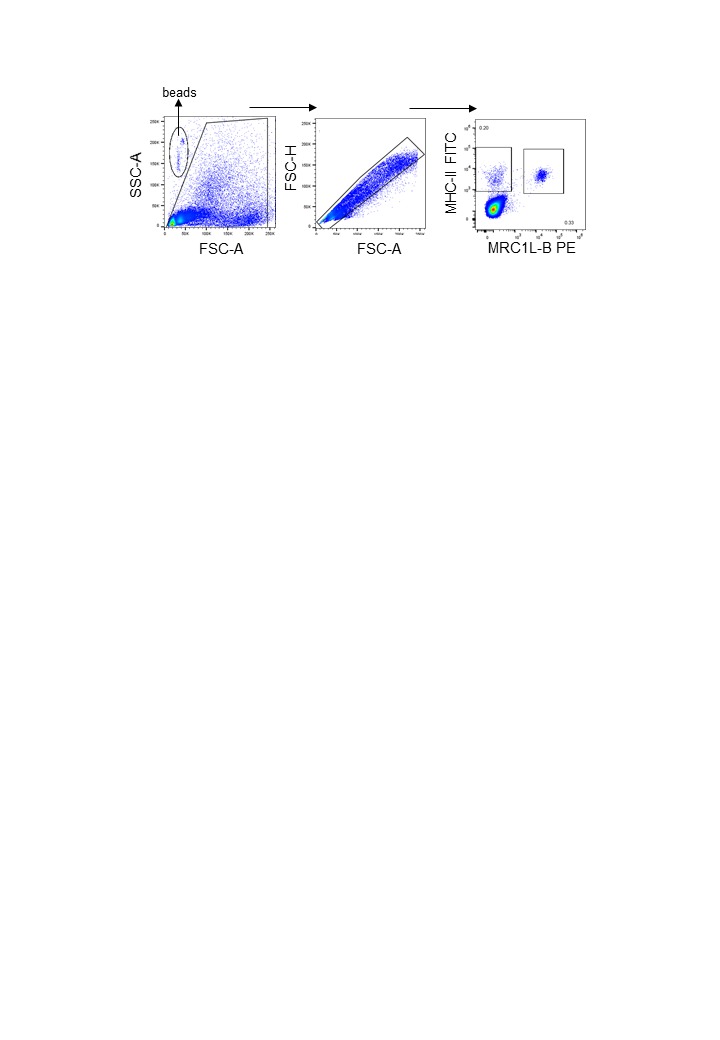


**Additional file 3. Gating strategy used to analyse whole blood samples.**

Supplement: Supplementary file 3 — Additional file 3. Gating strategy used to analyse whole blood samples. [file 13567_2020_840_MOESM3_ESM.docx]
